# *Lasiodiplodia theobromae* keratitis: A rare tropical fungal keratitis in a non-tropical climate

**DOI:** 10.1016/j.ajoc.2023.101944

**Published:** 2023-10-12

**Authors:** Calvin Hamerski, Alan D. Proia

**Affiliations:** a Campbell University Jerry M. Wallace School of Osteopathic Medicine, Lillington, NC, 27546, USA; b Departments of Pathology and Ophthalmology, Duke University Medical Center, Durham, NC, 27710, USA

**Keywords:** *Lasiodiplodia theobromae*, Cornea, Keratitis, Fungal keratitis, Geographic distribution, Case report

## Abstract

**Purpose:**

We present the clinical and histopathological findings of a geographically unique *Lasiodiplodia theobromae* fungal keratitis case in North Carolina. *L*. *theobromae* is a rare cause of fungal keratitis, and all but one of the 51 previously reported cases have occurred in patients living in the tropics.

**Observations:**

A man in his early 50s developed *L. theobromae* keratitis after being struck in the left eye by a piece of debris while using a flexible-cord weed trimmer. Intracapsular lensectomy and penetrating keratoplasty were necessary when initial antimicrobial therapy was ineffective. The best-corrected visual acuity was 20/40 four years postoperatively.

**Conclusions and Importance:**

Our patient is only the second example of *L. theobromae* keratitis in a patient living in a sub-tropical climate and the first case in the U.S.A. outside of Florida. Additional in-vitro antibiotic sensitivity testing and documentation of more clinical cases are needed to define the optimal therapy for *Lasiodiplodia theobromae* keratitis.

## 1 Introduction

*Lasiodiplodia theobromae*, also known as *Botryodiplodia theobromae*, is a dematiaceous (pigmented) fungus common in the tropics,^1^ where it is a frequent wound parasite on plants such as bananas.^2^ In the southern United States, this fungus is a common cause of wood stain,^3^ and it is also associated with plant rot in cotton, citrus fruit, Honey Dew melons, and strawberries.^4^ *L. theobromae* is a rare cause of severe keratitis, sometimes requiring penetrating keratoplasty.^5^ Cases of endophthalmitis and panophthalmitis have been reported rarely.^6^, ^7^, ^8^

We present the successful treatment of a man from North Carolina with corneal infection by this unusual fungus. Our patient represents, to our knowledge, the first individual in the U.S.A. outside of Florida who has developed *L. theobromae* keratitis.

## 2 Case report

A man in his early 50s was struck in the left eye by a piece of debris while using a flexible-cord weed trimmer. An outside ophthalmologist evaluated him the next day for ocular discomfort and foreign body sensation. Visual inspection disclosed an epithelial infiltrate with putative organic material at its base in the left paracentral cornea. The foreign material was extracted, and the eye was patched after applying 3.5 mg/gm neomycin/10,000 U/mg polymyxin B sulfate/0.1 % dexamethasone (Maxitrol®) ointment. Topical ciprofloxacin was started two days due to the persistence of the epithelial infiltrate and inflammation. There was a slight blurring of his vision, and the best corrected visual acuity in the left eye was 20/40. Intraocular pressure was 8 mmHg O.D. and 13 mmHg O.S. Therapy with ciprofloxacin was ineffective, and 15 days after injury there was stromal opacification superocentrally that appeared to penetrate the deep stroma. There was a small area of white deposit on the endothelium, along with one or two cells in the anterior chamber.

Over the next month, the patient experienced waxing and waning left eye pain and a scratching sensation. He was treated with atropine and various topical antimicrobials/antifungals, including ampicillin, ciprofloxacin, chloramphenicol, and natamycin. His care during this month also included two anterior chamber taps, from which cultures were negative. At the end of the second month following his injury, there was swelling and erythema of the left eyelids, increasing injection of the conjunctiva, and a hypopyon. He was referred to Duke University for further evaluation and treatment.

Approximately two months after the original injury best corrected visual acuity was 20/20 O.D. and 20/200 O.S. Intraocular pressure was 12 mmHg O.D. and 16 mmHg O.S. Slit lamp biomicroscopy found superficial punctate keratopathy, a superficial scar with deep corneal infiltrate, a plaque on the endothelium, and a small hypopyon. There was no epithelial defect at this time, and the vitreous and retina appeared unremarkable. The clinical impression was deep fungal keratitis. An anterior chamber tap and biopsy of the endothelial plaque were done that day. Gram stain of the aqueous sample showed rare leukocytes, but no organisms. Potassium hydroxide (KOH) preparation of the sample was negative, as were fungal, bacterial, and acid-fast bacilli cultures. Cytological examination of the endothelial plaque was negative for both organisms and malignancy. The patient was started on topical natamycin and amphotericin B, and oral ketoconazole, but the endothelial plaque persisted.

Two months later, there were keratic precipitates and filaments extending from the posterior cornea into the anterior chamber. Despite the persistence of the endothelial plaque and presumed fungal extension, visual acuity improved during this time and stabilized at 20/60 O.S. A fourth anterior chamber tap was performed four months following the original corneal injury. KOH/calcofluor white microscopic examination was negative, and the remainder of the fluid was cultured dropwise without streaking onto six agar plates and one agar slant. Three of the drops grew *Lasiodiplodia theobromae,* and the other four drops showed no growth.

**Fig. 1:**
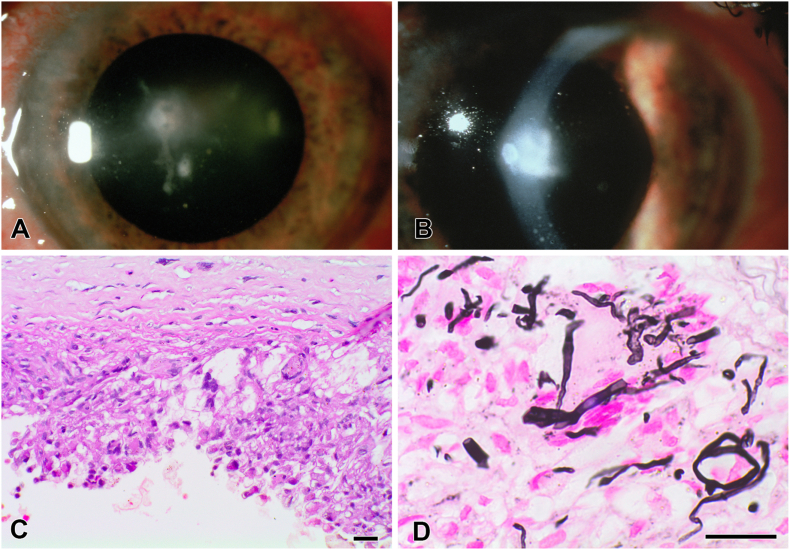
**A.** Clinical photograph three weeks prior to corneal transplantation. The central stroma has a dense white infiltrate in the deep layers of the cornea. **B.** Slit-lamp photograph three weeks prior to corneal transplantation. The slit beam illuminates a dense white endothelial plaque with extension into the anterior chamber. Endothelial keratic precipitates are visible on the inferior cornea. **C.** Photomicrograph demonstrating granulomatous inflammation involving the posterior corneal stroma in a region where Descemet's membrane is absent (H&E, magnification bar = 100 μM). **D.** Photomicrograph showing irregular hyphae within the granulomatous inflammatory infiltrate (methenamine silver, magnification bar = 100 μM).

Pharmacological management was changed to topical amphotericin B, natamycin, and flucytosine, and subconjunctival miconazole based on the identification of the fungus as *L. theobromae*. The endothelial plaque persisted, and at five months post-injury, an infiltrate was extending from the posterior cornea to the anterior lens capsule, with an accompanying decrease in visual acuity to 20/400 O.S. The patient was started on oral flucytosine, along with his ocular medications; visual acuity slowly improved, and the infection appeared to also improve and stabilize but not resolve. Examination of the left eye revealed 1 to 2+ injection of the conjunctiva, a central corneal scar with several endothelial deposits and adjacent keratic precipitates (Fig. 1A and B), a 1 mm fluffy deposit on the lens capsule with filaments extending into the anterior chamber, and several smaller deposits on the lens capsule. The lens was otherwise clear. At six months following the injury, additional lenticular involvement prompted a penetrating keratoplasty, intracapsular cataract extraction, and anterior vitrectomy. Postoperatively, the patient received topical amphotericin B and flucytosine, and oral itraconazole. No recurrence of the infection occurred, and the antifungals were discontinued after three months.

Pathological examination of the corneal button disclosed granulomatous inflammation near the center of the cornea in a region where Descemet's membrane was discontinuous (Fig. 1C). The inflammatory infiltrate contained macrophages and a few multinucleated giant cells; it involved the most posterior stroma and the area where Descemet's membrane would normally be present. Irregular hyphal fragments were seen in the section stained with methenamine silver (Fig. 1D) but not in a section stained with periodic acid-Schiff reagent. The hyphae were in the posterior stroma and within the adherent inflammatory infiltrate. The hyphae varied in width (4–6 μM), creating irregular contours. Septa were seen in only an occasional fragment. No fungi were identified in the lens, presumably because of its removal using a cryoprobe.

The patient experienced graft rejection fourteen months after the penetrating keratoplasty, which was successfully treated with aggressive steroid therapy. He underwent a wound revision four months post-op, and eight months post-op required a secondary posterior chamber intraocular lens placement. Eighteen months later, he experienced an inferior rhegmatogenous retinal detachment in his left eye, treated with a scleral buckle procedure, external retinal cryopexy, and drainage of subretinal fluid. Four years after discontinuing post-op antifungal therapy, his best corrected visual acuity in the left eye was stable at 20/40.

## 3 Discussion

**Fig. 2:**
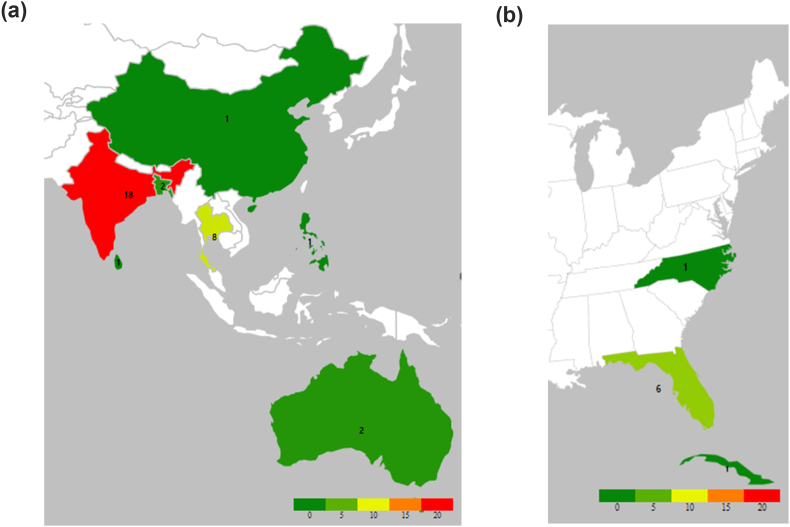
Heat map showing reported cases of L. theobromae ocular infections in East Asia, Australia (**A**), and the east coast of the United States, including our patient, and Cuba (**B**). Reported cases elsewhere are not shown for legibility (see Table 1).

*Lasiodiplodia theobromae* is a common fungus but a relatively rare cause of keratitis, with only 51 cases identified in our literature review (Table 1). Most of these cases were from the tropics or south Florida (Fig. 2, Table 2), which is reasonable since the fungus is most abundant in these climates.^2^ Our case is unusual due to its occurrence in a more temperate climate and represents the first reported case in the United States outside of Florida. North Carolina has a sub-tropical climate, and *Lasiodiplodia* keratitis in such a climate seems exceptionally rare, with one case reported in Hong Kong.^9^ The patient's initial treatment with Maxitrol® ointment may have facilitated his developing *Lasiodiplodia* keratitis since corticosteroids^10^ are contraindicated in cases of corneal foreign bodies^11^^,^^12^ until fungal infection is excluded.^13^

**Table 1:** Previous *Lasiodiplodia theobromae* keratitis cases.

First Author	# of Cases	Location	Year	Treatment	Outcome(s)/Visual Acuity
Puttanna^20^	2	India	1967	Medical, Medical	Perforated (N.L.P.), Perforated (N.L.P.)
Laverde^21^	1	Colombia	1973	Cauterization	“Healed after 20 days.”
Valenton^22^	1	Philippines	1975	Medical	LP
Rebell^18^	4	Florida/Cuba	1976	Medical x3, PKP x1	20/30+, 20/25, 20/20, 20/50
Liesegang^23^	1	Florida	1980	–	–
Dutta^24^	1	India	1981	–	–
Slomovic^8^	1	Florida	1985	Medical	Enucleation (N.L.P.)
Thomas^25^	1	India	1991	P.K.P.	“Graft clear.”
Gonawardena^26^	1	Sri Lanka	1994	–	–
Rosa^27^	1	Florida	1994	–	–
Dunlop^28^	2	Bangladesh	1994	–	–
Hagan^29^	6	Ghana	1995	–	–
Srinivasan^30^	6	India	1997	–	–
Borderie^6^	1	Guyana	1997	Lensectomy	Enucleation (N.L.P.)
Gopinathan^31^	7	India	2002		
Donnio^7^	1	Martinique	2006	Medical	Evisceration (N.L.P.)
Thew^32^	2	Australia	2008	–	–
Saha^19^	1	India	2012	P.K.P.	“Graft failed after 3 months.”
Samudio^33^	1	Paraguay	2014	PKP	–
Lekhanont^5^	2	Thailand	2015	Medical, PKP	20/200, counting fingers
Li^9^	1	Hong Kong	2016	PKP	6/30
da Rosa^17^	1	Brazil	2018	PKP	Hand movements
Tangmonkongvoragul^34^	6	Thailand	2021	PKP or patch graft (5/6)	–

- No data available; LP – light perception; NLP – no light perception; PKP – penetrating keratoplasty.

**Table 2:** Geographic distribution of 51 previously reported cases of Lasiodiplodia theobromae keratitis.

Country/# of cases		Country/# of cases	
India	18	Colombia	1
Thailand	8	Cuba	1
Ghana	6	Guyana	1
U.S.A. (Florida)	6	Martinique	1
Australia (Queensland)	2	Paraguay	1
Bangladesh	2	Philippines	1
Brazil	1	Sri Lanka	1
China (Hong Kong)	1		

Our patient's *Lasiodiplodia theobromae* remained undetected by culture and microscopy until four months after the corneal injury. *L. theobr*omae keratitis in previously reported cases has not been occult on culture and microscopy, with many infections diagnosed during initial corneal scraping. Our patient did not have corneal scrapings, as there was no apparent superficial involvement by the infection. Instead, pathogen identification was attempted using cultures of aqueous humor from anterior chamber taps. From the progression of slit-lamp findings in this case, we speculate that the inciting trauma seeded the deep corneal stroma with the fungus, which then spread posteriorly into the anterior chamber. A low fungal load likely caused the negative aqueous humor cultures until late in the case. Biopsy of the sub-endothelial plaque also noted no microbes on culture and microscopy, most likely due to the samples being inflammatory infiltrate devoid of organisms. Possibly, a suture pass through the deep corneal infiltrate^14^ together with next generation sequencing and metagenomics analysis^15^^,^^16^ may have diagnosed the infection sooner.^14^, ^15^, ^16^

Pre-operative antimicrobial management (topical natamycin, amphotericin B, and flucytosine; oral ketoconazole and flucytosine; and subconjunctival miconazole) failed to control the infection in our patient. Oral itraconazole, along with topical amphotericin B and flucytosine, prevented postoperative recurrence of the infection. *Lasiodiplodia* keratitis in the literature has shown a response to natamycin, amphotericin B, and, more recently, voriconazole.^5^^,^^9^^,^^17^, ^18^, ^19^ In vitro antifungal testing in two cases showed a sensitivity of *L. theobromae* to both amphotericin B and voriconazole,^17^^,^^19^ and voriconazole has had varying degrees of success in four *L. theobromae* keratitis cases.^5^^,^^9^^,^^19^ However, voriconazole was not an option for our patient's treatment since it had not yet been approved for medical use. Amphotericin B has played a role in successful treatment in multiple cases, though never as monotherapy.^5^^,^^17^^,^^19^

After the identification of *L. theobromae* in our case, miconazole and itraconazole were tried in succession due to their extended spectrum relative to other azole drugs. While miconazole was ineffective, itraconazole was part of the polytherapy that prevented recurrence postoperatively. It is unclear if the addition of itraconazole contributed to this success or if the decreased postoperative fungal load allowed clearance by another drug or the patient's immune system. In vitro testing in one article, published after our case, showed one *L. theobromae* strain to be resistant to itraconazole.^17^ Literature on the use of itraconazole against this organism is otherwise scarce. Indeed, the efficacy of any one drug against *L. theobromae* is challenging to discern in the literature. Further in-vitro sensitivity testing and documentation of more clinical cases are needed.

## Ethics approval and consent to participate

This case report was approved by the Duke Health Institutional Review Board (protocol number: Pro00111329). Collection and evaluation of protected health information complied with the Health Insurance Portability and Accountability Act, and the manuscript adhered to the tenets of the Declaration of Helsinki.

## Funding

There is no funding or grant support for this case report.

## Authors’ contributions

Both authors attest that they meet the current ICMJE criteria for Authorship.

## Consent for publication

Not applicable; this case report does not include any personally identifiable information.

## Declaration of competing interest

The authors declare that they have no known competing financial interests or personal relationships that could have appeared to influence the work reported in this paper.
